# Study on the Photocatalytic Properties of Flower-Shaped SnO_2_

**DOI:** 10.3390/nano12193419

**Published:** 2022-09-29

**Authors:** Tingting Shao, Xinrui Cao, Juntang Dong, Jing Ning, Fuchun Zhang, Xiaoyang Wang, Yuyang Cheng, Huirong Kou, Weibin Zhang

**Affiliations:** 1 School of Physics and Electronic Information, Yan’an University, Yan’an 716000, China; 2 School of Physics and Optoelectronic Engineering, Yangtze University, Jingzhou 434023, China

**Keywords:** SnO_2_, flower shape, photocatalytic properties, methyl orange

## Abstract

Using cetyltrimethylammonium bromide (CTAB) as the surfactant from the precursors of SnCl_2_·2H_2_O, the flower-shaped nano composite of tin oxide (SnO_2_) is prepared by the simple eco-friendly hydrothermal method. We can see that the as-prepared SnO_2_ sample has a rutile phase crystal structure with regular-shaped nanosheets, and the nanosheets were cross-assembled to form nanoflowers. The band gap of the as-prepared SnO_2_ sample is 2.26 eV, which is close to the calculated energy gap of 2.58 eV based on density functional theory. The sample is used to degrade the organic dye, and this preliminary application study indicates that the as-prepared SnO_2_ sample has good stability and reusability in the visible light assisted degradation of methyl orange. Through capture experiments, it is determined that electrons and holes play a major role in the degradation process. The reaction mechanism is also analyzed to indicate the internal relationship between the as-prepared SnO_2_ samples and its photocatalytic properties.

## 1. Introduction

At present, water pollution is a direct threat to human survival, and it is a big problem that must be addressed. In water pollution control, wastewater containing dyes is a challenge for wastewater treatment due to its variety, high toxicity, severe environmental impact, and difficulty in degradation [1]. Photocatalytic technology is an efficient, energy-saving, and non-cross-contaminating method to break down organic pollutants contained in dyes in wastewater into non-toxic or less-toxic small molecules [2]. Therefore, the preparation of excellent photocatalysts is very important in the treatment of wastewater containing dyes.

SnO_2_ is an inexpensive, non-toxic, high electron mobility, and high photosensitivity compound, which has attracted wide attention in basic research and practical applications, such as photocatalysts, gas sensors, solar cells, electrode materials, and so on [3]. By modulating the morphology of the SnO_2_ nanocrystals, the physical and chemical properties can be manipulated [4]. In recent years, various morphologies of SnO_2_ have been prepared, including nanorods, nanowires, nanobelts, and nanoflowers [5,6,7,8]. Three-dimensional SnO_2_ hierarchical nanostructures are beneficial to improving its performance, due to a low density, high porosity, high specific surface area, and so on [9]. Acharyulu et al. synthesized TiO_2_/SnO_2_ hollow spheres via a hydrothermal process and the samples can be used for photocatalytic mineralization of methylene blue (MB), methyl orange (MO), rhodamine B (RhB), and Congo red (CR) dyes under UV exposure [10]. Lei et al. proposed a facile route to prepare a novel Fe_2_O_3_/SnO_2_ heterojunction structure, in which the nanobelt arrays grown on iron foil naturally form a Schottky-type contact and provide a direct pathway for the photogenerated excitons, with a degradation rate constant of the Fe_2_O_3_/SnO_2_ film of approximately 12 times that α-Fe_2_O_3_ nanobelt arrays [11]. Xu et al. synthesized SnO_2_@ZnO hierarchical nanostructures by a simple two-step microwave-assistant hydrothermal method, and remarkably enhanced the photocatalytic degradation of a MB aqueous solution under visible light irradiation [12]. Bezzerrouk et al. synthesized SnO_2_ thin film via an ultrasonic spray pyrolysis technique in the inner wall of a glass tube, with the SnO_2_ thin film presenting the best MB degradation efficiency compared to the other processes [13].

In this work, we used a simple method to synthesize hierarchical flower-like SnO_2_ nanostructures by the hydrothermal method. The properties of the product are investigated by using X-ray diffraction (XRD), scanning electron microscopy (SEM), transmission electron microscopy (TEM), X-ray photoelectron spectrum (XPS), as well as Raman and ultraviolet–visible (UV-Vis) spectra. In addition, the experiment shows that the SnO_2_ product exhibits a high catalytic activity, good stability, and reusability for degradation of MO under visible light.

## 2. Experimental

### 2.1. Synthesis of SnO_2_

In this paper, the chemical precursors were all analytical grade. SnO_2_ was prepared by a facile one-step hydrothermal method as follows: 1 g SnCl_2_·2H_2_O was first dissolved in 40 mL deionized water ultrasonically for 5 min, and stirred for 30 min to form the precursor solution; 1.6467 g CTAB was dissolved in 40 mL deionized water, with ultrasonic treatment for 15 min; and 0.7293 g NaOH was added and stirred for 30 min. After being fully dissolved, we added the precursor solution. The mixture solution was stirred for 60 min, and then transferred to a 100 mL Teflon-lined stainless-steel autoclave and kept at 140 °C for 12 h. After the reaction, the precipitate was washed with anhydrous ethanol and deionized water for several times. Finally, light-yellow SnO_2_ powder was obtained after drying at 80 °C for 8 h. (Figure S1 shows the SnO_2_ product synthesis procedure).

### 2.2. Photocatalytic Testing

In this work, MO was selected as the target pollutant to evaluate the photocatalytic activity. Specifically, 50 mg of the catalyst was added to a 10 mg/L MO solution, and these solutions were stirred in the dark for 30 min to make the photocatalyst reach the adsorption–desorption equilibrium of MO. The light source used for photodegradation of the MO solution was a 500 W xenon lamp. The degradation reaction was carried out under stirring conditions at various times points: 0, 20, 40, 60, 80, 100, and 120 min. A 5 mL solution was taken out each time for centrifugation to completely precipitate, and then their respective supernatants were taken. The corresponding absorbance ABS value (absorption wavelength is 300 nm–600 nm) was tested using an ultraviolet spectrophotometer, with a residual degradation ratio of MO: η = C_t_/C_0_, where C_0_ (mg/L) and C_t_ (mg/L) represent the initial concentration of the dye and the mass concentration of the dye solution at time t, respectively. Additionally, a SnO_2_ adsorption study was carried out in the contaminated solutions for 2 h, and a pollutant circulation photolysis study was carried out for 10 h under visible light.

## 3. Results and Discussion

### 3.1. Materials Characterization

**Figure 1:**
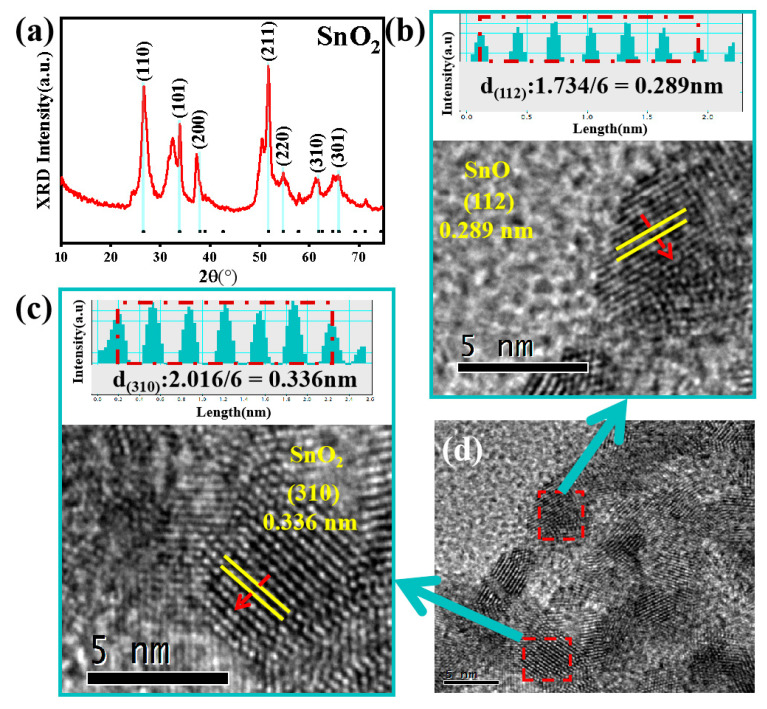
XRD patterns of the as-prepared SnO_2_ (**a**); TEM image of SnO_2_ (**b**–**d**). Inset: enlarged view of the surface (**b**,**c**), and a low magnification TEM image (**d**).

The crystal phases of the as-prepared SnO_2_ products were characterized by XRD and the results are shown in Figure 1a. The scan range is from 10° to 75°. By comparing the SnO_2_ PDF standard card (JADES: 71-0652), the 2θ values at 26.5°, 34°, 37.8°, 51.9°, 54.8°, 64.7°, and 65.9°correspond to the crystalline planes (110), (101), (200), (211), (220), (310), and (301), which can determine the crystal structure of the SnO_2_ is in the rutile phase based on the 71-0652 file of the JCPDS [14,15]. However, there are two miscellaneous peaks generated around 32° and 50.1°, which may result from SnO generated during the preparation process. Figure 1d exhibits the high-resolution transmission electron microscopy (HRTEM) image of the as-prepared SnO_2_. The visible lattice fringe with an interplanar crystal spacing of 0.289 nm, shown in Figure 1b, is assigned to the (112) plane of SnO, which can confirm the source of the XRD peaks at 32° and 50.1°. The lattice fringe of the crystal spacing of 0.336 nm, shown in Figure 1c, corresponds to the (310) surface of SnO_2_. Besides the two peaks at 32° and 50.1°, there is no other obvious impurity peaks, and the diffraction peaks are sharp and strong, which is basically consistent with the results of the standard card, indicating that the synthesized SnO_2_ sample has good crystallinity.

**Figure 2:**
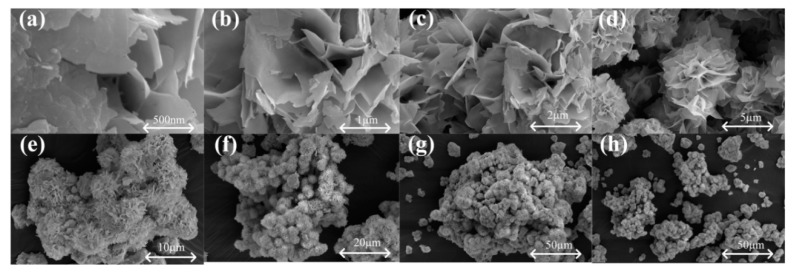
SEM images of the as-prepared SnO_2_: High magnification SEM images (**a**–**d**); Low magnification SEM images (**e**–**h**).

Figure 2 displays the SEM images of the as-prepared SnO_2_ sample, and the product has a flower-shaped morphology. The general morphology of the SnO_2_ product is shown in Figure 2e–h, and we can see that the products have a high yield and uniformity. As shown in Figure 2d, the SnO_2_ presents a uniform, flower-like architecture, with a diameter of approximately 8–9 μm. As we can see from Figure 2a–d, the SnO_2_ nanoflowers are constructed by a lot of nanosheets, which are self-assembled densely together and the surface is very smooth.

**Figure 3:**
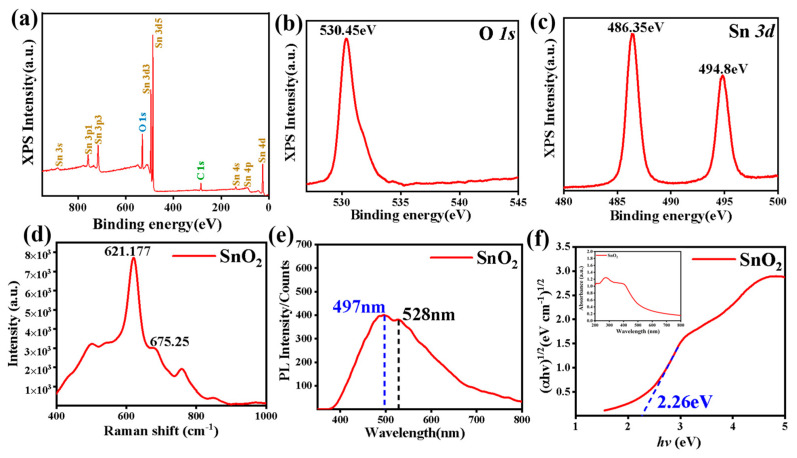
XPS spectra of as-prepared SnO_2_: full spectra (**a**); O 1s (**b**); Sn 3d (**c**); Raman spectra of the as-prepared SnO_2_ (**d**); PL spectra of the as-prepared SnO_2_ (**e**); Band gap of as−prepared SnO_2__,_ the insert figure is UV-Vis absorption spectrum and (αhv)1/2-hv curves (**f**).

Furthermore, the chemical compositions and valence states of the SnO_2_ samples were analyzed by XPS. High-resolution XPS analysis was performed for each element and calibrated using the C 1s standard carbon peak. Figure 3a shows the wide range of XPS spectra of the as-prepared SnO_2_ product. In Figure 3b,c, no peaks other than Sn, O, and C can be identified. The presence of a C element was observed, which may be due to the pollution of the sample surface by carbon materials. Figure 3b shows the XPS spectra of O 1s. The peak at 530.45 eV corresponds to O 1s, and the binding energy indicates that the oxygen atoms exist as O^2−^ species in the product. Figure 3c shows the high-resolution XPS spectra of Sn 3d, in which the peaks at 486.35 eV and 494.8 eV are from Sn 3d_5/2_ and Sn 3d_3/2_, respectively. The result is in agreement with the reported values in the literature [16]. XPS results confirmed that the synthesized product is pure SnO_2_ in terms of elements. Thus, the SnO_2_ flower-like nanostructures were determined to be successfully synthesized through the above analysis.

In order to clarify the effect of the as-prepared SnO_2_ products on the fluctuation of chemical bonds, the molecular vibration information of the specimens was characterized by a Raman spectrometer and the result is shown in Figure 3d. For the as-prepared nanoflower SnO_2_, the main peaks are concentrated at 621.177 cm^−1^ and 675.25 cm^−1^. The peak at 621.177 cm^−1^ is attributed to the A_1g_ vibration mode of SnO_2_ with a tetragonal rutile structure. The peak at 675.25 cm^−1^ is induced by the size effect of the very small SnO_2_ nanocrystals and in fact it does not appear in bulk SnO_2_. PL measurement is powerful to study the recombination rate of the *e^−^* and *h^+^* pair in the samples, and the recombination rate of the *e^−^* and *h^+^* pair is proportional to the PL intensity. Figure 3e shows the emission spectra of the as-prepared SnO_2_ products, and the PL spectrum is carried out in the wavelength range of 350—800 nm using an excitation wavelength of 325 nm. The as-prepared SnO_2_ products show bands located at 497 nm and 528 nm. The stronger peak is present at 497 nm, which is due to the recombination of the electron–hole pairs, and the peak value in the spectra maybe also related to the lifetime of the excited electrons before recombination. The stronger the emission intensity of the PL spectrum, the larger the recombination rate of the electrons and holes, and then the lower the photocatalytic rate [17].

Figure 3f shows the UV-Vis absorption spectrum of the as-prepared SnO_2_. As we can see from Figure 3f, the spectrum of the as-prepared SnO_2_ shows the highest peak at about 290 nm, which indicate the as-prepared SnO_2_ can be excited by a small part of the visible spectrum, and it is mainly excited by ultraviolet light. Further, to investigate the band gap of SnO_2_, the UV-Vis diffuse reflectance spectrum (DRS) was obtained with the help of the Kubelka–Munk formula (Equation (S1)) [18], indicating that the SnO_2_ displayed a band gap value of 2.26 eV. Moreover, the valence band (VB) and conduction band (CB) position was obtained from (Equations (S2)–(S4)), as proposed by Ralph G. Pearson in 1988 [19]. Thus, the E_CB_ values of SnO_2_ was calculated to be 0.65 eV, while the corresponding E_VB_ values was 2.91 eV, respectively.

From Figure 3f, we can see the band gap of the as-prepared SnO_2_ is 2.26 eV, which indicates that the material is a wide band gap semiconductor material. However, the band gap is much shorter than those of 3.2 and 3.6 eV reported by other teams [20,21]. We can infer that the as-prepared SnO_2_ may effectively degrade pollutants, because the wavelength range of the samples with a narrow band gap is generally wide.

### 3.2. Photocatalysis Performance

**Figure 4:**
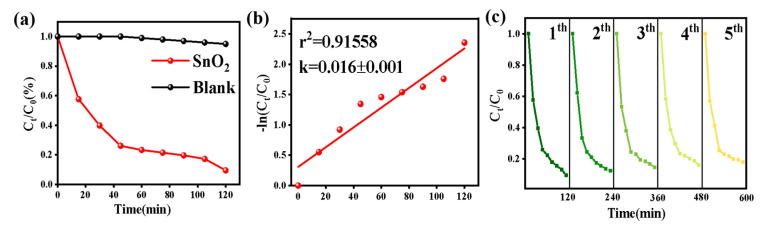
MO concentration (*C_t_/C*_0_) against photo degradation time (**a**); the fitting parameter for the pseudo-first-order kinetic curve (**b**); and the cycling performance test (**c**).

In order to evaluate the photocatalytic activity of the as-prepared SnO_2_, the photocatalytic performance of the aqueous MO solution was tested in the presence of the synthesized SnO_2_ product under visible light irradiation. The results are shown in Figure 4a. The blank test shows that light has little influence on the MO aqueous solution. However, when the solution is irradiated for 120 min in the presence of the as-prepared SnO_2_ product, the solution has almost no color. By processing the data, we obtained that the degradation rate of the as-prepared SnO_2_ to MO is 90.6%.

Further, kinetic curves were provided, with correlation coefficients (r^2^) over the value of 0.90, to study the photocatalytic process of the MO, indicating that all catalysts match the pseudo-first-order kinetic model in Figure 4b. The stability and reusability of the as-prepared SnO_2_ products were investigated by reusing the products. From Figure 4c, we can see that the activity of the as-prepared SnO_2_ products did not decrease significantly after five cycles of MO photo degradation under visible light irradiation.

### 3.3. Reaction Mechanism

**Figure 5:**
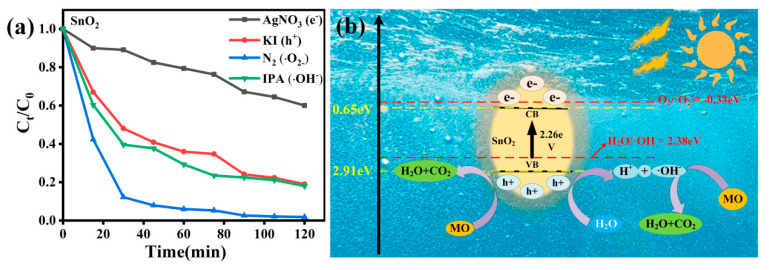
The influence of different scavengers on the photocatalytic degradation of MO by the as−prepared SnO_2_ (**a**); the reaction mechanism of the as−prepared SnO_2_ (**b**).

In order to study the degradation mechanism of the as-prepared SnO_2_ products, free-radical-capture experiments were carried out. In the photocatalytic process, because the super-oxide radicals (•O_2_*^−^*)*,* holes (*h^+^*)*,* and hydroxide radicals (•OH^−^) are considered as the most active intermediate free-radical species, they can directly transfer the radical unpaired electrons from the valence band maximum (VBM) to the identified lowest unoccupied molecular orbital (LUMO) [22]. These kinds of radical scavenger experiments on the degradation of MO are carefully carried out to inspect the underlying photo-degradation mechanism. A total of 0.3 mmol nitrogen (N_2_), 0.3 mmol potassium iodide (KI), 0.3 mmol argentum nitricum (AgNO_3_), and 0.3 mmol isopropanol (IPA) were added to the reaction system as the scavenger of •O_2_^−^, h^+^, e^−^, and •OH^−^, respectively. Figure 5a shows the change in the degradation kinetics in the presence of those above scavengers. We can see that under visible light irradiation, e^−^, •OH^−^, and h^+^ play predominant roles in the reaction.

The reaction mechanism of the as-prepared SnO_2_ products in the degradation of MO dyes is showed in Figure 5b. When MO and the as-prepared SnO_2_ solutions are irradiated with visible light, because the band gap of the as-prepared SnO_2_ is 2.26 eV, the SnO_2_ product absorbs energy and the electrons in the valence band (VB) are excited to jump to the conduction band (CB), and photogenic holes *h^+^* are left. Because the nanoparticles are in the aqueous phase and *h^+^* have strong oxidability, the H_2_O molecules adsorbed on the surface of the tin oxide can be oxidized to (*•*OH^−^) with high activity and can be directly degraded by reaction with MO. The reduction potential of the photocatalyst at the conduction band (0.65 eV) is lower than the reduction potential of O_2_ (−0.33 eV); so, the (•O_2_^−^) production conditions cannot be met. The (•O_2_^−^) cannot effectively decompose the organic dye, which is consistent with the captured experimental results. With their powerful oxidability, h^+^ and (•OH^−^) can effectively decompose the organic dyes [23]. The main equations are shown in (Equations (S5)–(S7)). Furthermore, due to the high surface area of the as-prepared SnO_2_, there are numerous active sites, which is good for adsorbing the active group.

### 3.4. Theoretical Calculation

**Figure 6:**
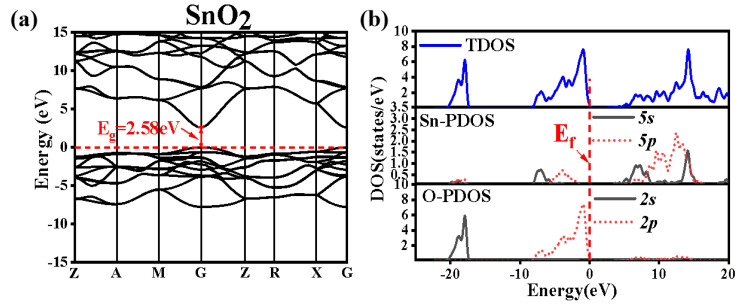
Band structure of pure SnO_2_ (**a**); the TDOS and PDOS of pure SnO_2_ (**b**).

In order to indicate the internal relationship between the photocatalytic activity and electronic structure of the as-prepared SnO_2_ products, the electronic structures of the pure SnO_2_ were calculated based on density functional theory (DFT), and the exchange interaction and related potential between the electrons were rectified by Becke’s Three Parameter Hybrid Functional using the LYP Correlation Functional (B3LYP). The band structure, the total, and part of the density of states (TDOS/PDOS) of the rutile-type SnO_2_ are shown in Figure 6.

In Figure 6a, the Fermi level is shown with a dashed line and assigned a zero on the energy scale. The occupied state below the Fermi energy is the valence band, and the unoccupied state lying above the Fermi energy is the conduction band. The conduction band minimum and the valence band maximum are located at the same G point, which demonstrate that SnO_2_ is a direct band-gap semiconductor. The Fermi level is close to the valence band, which means that the conductivity of SnO_2_ is lower. The calculated band gap value is 2.58 eV, which is less than the experimental value of 3.6 eV [24], but is very close to the UV-Vis spectrum-measured band gap of the as-prepared SnO_2_ of 2.26 eV. The exciting electrons in the valence band transfer to the conduction band through some distance, which may inhibit the recombination of electrons and holes. The band gap value can be used to show the performance of the excited electrons. If the band gap of catalyst is small, when the matched or greater energy of the electromagnetic wave is close to the catalyst surface, the electron–hole pairs can be easily generated, which is good for the dye photocatalytic degradation. That is maybe the reason why the MO can be degraded under visible light by our as-prepared SnO_2_; in other reports, the dye degradation is under ultraviolet light.

Figure 6b shows the DOS and PDOS of the rutile-type SnO_2_. There are three parts of the total density state, from the −20 eV to −15.00 eV region, which is far from the Fermi level, and is dominated by the O 2*s^2^* states. Due to the deep-level orbital role, the analysis of this region is unnecessary. Generally, the electrical properties of a semiconductor are determined by the electron states near the Fermi surface, and only the electrons near the Fermi surface can possibly jump into the nearby empty states. The −8.00 eV to 0 eV region is dominated by O *2p* states and a few Sn *5s*, Sn *5p* states, and especially around the Fermi energy level, the DOS is mainly from the O *2p* states. The conduction band is mainly dominated by Sn *5s* Sn *5p* and a few O *2p.*

## 4. Conclusions

In this work, flower-shaped SnO_2_ was successfully synthesized by a one-step hydrothermal method. The XRD, Raman, and SEM examinations revealed that the as-prepared SnO_2_ products possess pure-phase, good crystallinity. In the visible light range, the degradation rate of MO was 90.6%, and the SnO_2_ can keep a high degradation efficiency after five cycles, which indicate its potential application in eliminating organic pollutants. Through free-radical-capture experiments, we found that the electrons and holes play major roles in the degradation process. The band gap of the as-prepared SnO_2_ measured by the UV-Vis spectrum was 2.26 eV, which is close to the calculated band gap value of 2.58 eV based on DFT. Such a flower-shaped structure is also expected to present good performance in gas sensing.

## Data Availability

All data are presented in the form of charts in the article.

## Supplementary Materials

The following supporting information can be downloaded at: https://www.mdpi.com/article/10.3390/nano12193419/s1, Figure S1: The diagram of SnO_2_ product synthesis procedures [25,26,27,28,29,30].
