# Supplementary material for: Study on the Photocatalytic Properties of Flower-Shaped SnO2
Source: Nanomaterials (Basel). 2022 Sep 29;12(19):3419. doi: 10.3390/nano12193419 (PMC9565917; doi:10.3390/nano12193419)
Supplement: Supplementary file 1 [file nanomaterials-12-03419-s001.zip › nanomaterials-1895362-supplementary.pdf]

nanomaterials

Supporting Information

Study on the Photocatalytic Properties of Flower-Shaped SnO_2

Tingting Shao [1], Xinrui Cao [1], Juntang Dong [1], Jing Ning [1], Fuchun Zhang [1,*], Xiaoyang Wang [1], Yuyang Cheng [1], Huirong Kou [1] and Weibin Zhang [2]

[1] School of Physics and Electronic Information, Yan'an University, Yan'an 716000, China
[2] School of Physics and Optoelectronic Engineering, Yangtze University, Jingzhou 434023, China
* Correspondence: zhangfuchun72@163.com

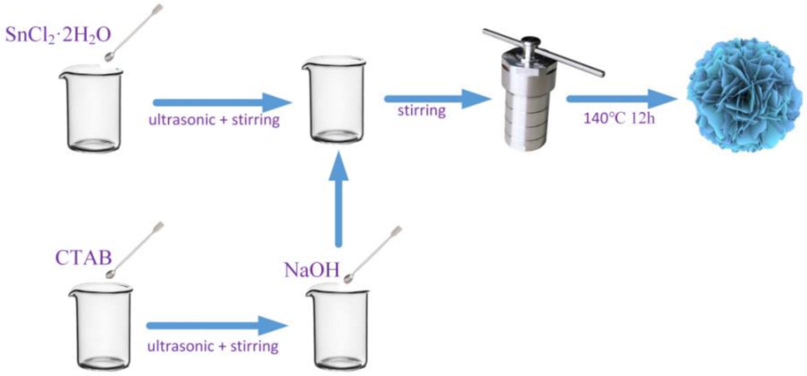

Figure S1. The diagram of SnO_2 product synthesis procedures.

Chemicals and materials

Tin (II) chloride dihydrate (SnO_2), Hexadecyl trimethyl ammonium Bromide (CTAB) and Sodium hydroxide (NaOH) were purchased from Shanghai Yien Chemical Technology Co., LTD. Ethanol absolute were supplied by Tianjin Kemiou Chemical Reagent Co., LTD. All of these materials were analytically pure and utilized directly.

Band scheme confirmation

Energy band gap (E_g) about pure SnO_2 was calculated according to the following Kubelka- Munk formula:

$$\alpha h v = A(hv - E_g)^{1/2} \quad \text{(Equation S1)}$$

where a, h, v and A are the absorption coefficient, Planck's constant, the optical coefficient frequency and the proportionality constant[1-3].

The E_{VB} and E_{CB} values of SnO_2 was calculated using the following formula:

$$E_{VB}=\chi - Ee + 1/2E_g \quad \text{(Equation S2)}$$

$$E_{CB}=E_{VB} - E_g \quad \text{(Equation S3)}$$

χ corresponds to the electronegativity of semiconductors, and E_e may be 2.91eV. E_g refers to the band gap of a semiconductor. The χ values for SnO_2 can be obtained by the following function[4]:

$$\chi_{SnO2}=(\chi_{(Sn)}{}^{1}\chi_{(O)}{}^{2})^{1/3} \quad \text{(Equation S4)}$$

where χ represents the absolute electronegativity of elements, and the absolute electronegativity values of Sn and O are 4.30 eV and 7.59 eV[5-6] and the absolute electronegativity values of SnO_2 is 6.28 eV . Therefore, the E_{CB} values for SnO_2 was calculated to be 0.65 eV , while the related E_{VB} values was 2.91 eV , respectively.

The proposed reaction pathway for SnO_2.

$SnO_2+hv \rightarrow (e^-+h^+)$ (Equation S5)

$h^++H_2O \rightarrow H^++(\bullet OH^-)$ (Equation S6)

$h^+/\bullet OH^-+dye \rightarrow degradation+products$ (Equation S7)

Radical scavengers experiments

Nitrogen will be introduced into the bottom of the tube to create a uniform bubble to capture superoxide radicals. All the other conditions remain unchanged.

Characterization

The microstructure of the specimens was determined via X-ray diffraction (XRD) in the 2θ range of 10-80° by using the Cu target (λ, $K_{\alpha 1}$= 1.54056 Å) to generate X-ray radiation. The instrument was operated at 40 kV and 30 mA with a scanning rate of 6 °/min and a step size of 0.02°. Transmission electron microscopy (TEM) was employed to investigate the micromorphology and the microstructure by using an operating voltage of 200 kV. The morphology of the specimens was detected via scanning electron microscopy (SEM) with a 3.0 kV working voltage beam. The X-ray photoelectron spectroscopy (XPS) characterization of the specimens was performed on a photoelectron spectrometer. The UV-Vis absorption spectra of the specimens were recorded by using an ultraviolet-visible near-infrared spectrometer.

[1] Tong, H.; Ouyang, S. X.; Bi, Y. P.; Umezawa, N.; Oshikiri, M.; Ye, J. H. Nano-photocatalytic Materials: Possibilities and Challenges. *Advanced Materials* **2012,** *24* (2), 229-251.
[2] Wazir, M. B.; Daud, M.; Ali, F.; Al-Harthi, M. A. Dendrimer assisted dye-removal: A critical review of adsorption and catalytic degradation for wastewater treatment. *J Mol Liq* **2020,** *315*, 113775.
[3] Crini, G.; Lichtfouse, E. Advantages and disadvantages of techniques used for wastewater treatment. *Environmental Chemistry Letters* **2019,** *17* (1), 145-155.
[4] Korotta-Gamage, S. M.; Sathasivan, A. A review: Potential and challenges of biologically activated carbon to remove natural organic matter in drinking water purification process. *Chemosphere* **2017,** *167*, 120-138.
[5] Wacławek, S.; Silvestri, D.; Hrabák, P.; Padil, V. V. T.; Torres-Mendieta, R.; Wacławek, M.; Černík, M.; Dionysiou, D. D. Chemical oxidation and reduction of hexachlorocyclohexanes: A review. *Water Research* **2019,** *162*, 302-319.
[6] Talaiekhozani, A.; Talaei, M. R.; Rezania, S. An overview on production and application of ferrate (VI) for chemical oxidation, coagulation and disinfection of water and wastewater. *Journal of Environmental Chemical Engineering* **2017,** *5* (2), 1828-1842.
